# Acute Hepatic Injury Associated with Acute Administration of Synthetic Cannabinoid XLR-11 in Mouse Animal Model

**DOI:** 10.3390/toxics10110668

**Published:** 2022-11-06

**Authors:** Ayman Alzu’bi, Mazhar Salim Al Zoubi, Bahaa Al-Trad, Manal Isam AbuAlArjah, Malek Shehab, Hiba Alzoubi, Dima Albals, Gamal T. Abdelhady, Waseem El-Huneidi

**Affiliations:** 1 Department of Basic Medical Sciences, Faculty of Medicine, Yarmouk University, Irbid 211-63, Jordan; 2 Department of Biological Sciences, Faculty of Science, Yarmouk University, Irbid 211-63, Jordan; 3 Department of Medicinal Chemistry and Pharmacognosy, Faculty of Pharmacy, Yarmouk University, Irbid 211-63, Jordan; 4 Department of Anatomy and Embryology, Faculty of Medicine, Ain Shams University, Cairo 11566, Egypt; 5 Department of Basic Medical Sciences, College of Medicine, University of Sharjah, Sharjah 27272, United Arab Emirates

**Keywords:** synthetic cannabinoids, SCs, XLR-11, oxidative stress, acute liver injury, hepatic injury

## Abstract

The widespread recreational use of synthetic cannabinoids (SCs) has become a serious health issue. Reports of life-threatening intoxications related to SC consumption have markedly increased in recent years, including neurotoxicity, cardiotoxicity, nephrotoxicity, and hepatotoxicity. We investigated the impact of acute administration of the synthetic cannabinoid XLR-11 (3 mg/kg, i.p. for 5 consecutive days) on the liver in BALB/c mouse animal model. Using real-time quantitative RT-PCR, MDA assay, and TUNEL assay, we found consistent up-regulation of a variety of genes involved in oxidative stress (NOX2, NOX4, and iNOS), inflammation (TNF-α, IL-1β, IL-6), and apoptosis (Bax) in the liver of XLR-11 treated mice compared to control mice. These finding were supported with an elevation of MDA levels and TUNEL positive cells in the liver of XLR-11 treated mice which further confirm increased oxidative stress and apoptosis, respectively. Histopathological analysis of the liver of XLR-11 treated mice confirmed pronounced hepatic necrosis associated with inflammatory cell infiltration. Furthermore, elevated ALT and AST serum levels were also identified in XLR-11 treated mice indicating possible liver damage. Overall, SC-induced hepatotoxicity seems to be mainly mediated by activated oxidative stress and inflammatory processes in the liver, but the specific mechanisms involved require further investigations. However, the present study shed light on the potential deleterious role of acute administration of SCs in the progression to acute hepatic injury which enhances our understanding of the adverse effect of SC consumption.

## 1. Introduction

Synthetic cannabinoids (SCs) are a class of psychotropic drugs that are chemically designed to bind to the cannabinoid receptors 1 and 2 (CB1 and CB2) and mimic the effects of Δ9-tetrahydrocannabinol (THC), the main psychoactive ingredient of cannabis [1,2]. SCs are found in the illicit drug market with several street names such as Spice and K2, where they are deceitfully marketed as safer alternatives to marijuana. However, as most SCs are described as full agonists at CB1 and CB2, with higher potency and binding affinity than THC (partial agonist), it has been recently demonstrated that SCs may result in stronger psychoactive effects, as well as a higher risk of other adverse effects [3,4,5].

The expression of the cannabinoid receptors CB1 and CB2 is discovered in a variety of tissues throughout the human body. Under physiological settings, CB1 is substantially expressed in the central nervous system and lower but functionally significant in the periphery, whereas CB2 is largely expressed in immune cells and contributes to immune system modulation and in hematopoietic cells [6,7]. Exogenous stimulation of the cannabinoid receptors within the endocannabinoid system has been revealed to cause several deleterious side effects not only to the CNS, but also to many other systems [8,9].

SCs are now recognized to be the most linked with substantial health concerns among the wide range of regularly used novel psychoactive drugs found in illegal drug markets [8,9]. Anxiety, hallucinations, seizures, tachycardia, hypertension, vomiting, and nausea have all been reported as side effects of SC usage [10,11]. In particular, case reports have recently reported acute liver injury following the use of SCs; this includes patients without any prior history of liver disease [12]. Such patients were usually presented to the emergency departments with symptoms such as abdominal pain, vomiting, fatigue, and lethargy. Laboratory findings of those patients mostly revealed significantly elevated liver enzymes such as aspartate aminotransferase, alanine aminotransferase, alkaline phosphatase, and bilirubin [12,13,14,15,16].

XLR-11 is a synthetic cannabinoid that has a higher affinity for the CB2 receptor and is frequently found in herbal smoking mixtures all over the world. As opposed to the non-fluorinated equivalent, XLR-11 has a fluoride group at the end of the pentyl side chain, which boosts the molecule′s stability and lengthens the half-life while also increasing its affinity to the CB1 receptor. In vitro and in vivo studies by Wiley and colleagues (2013) further supported the cannabimimetic properties of XLR-11, demonstrating that this compound is a complete agonist of both the CB1 and CB2 receptors [17,18].

Despite of increasing concerns about the direct link between SC consumption and liver injury, the pathophysiology of SC-induced liver injury remains unknown. This study represents an in vivo assessment of SC-induced hepatotoxicity in mice. We investigated the potential deleterious effect of acute administration of the synthetic cannabinoid XLR-11 on the liver tissue. Its effect on gene expressions of specific markers of oxidative stress, apoptosis, inflammatory response, and fibrosis was investigated by real-time quantitative RT-PCR. Histopathological changes and level of liver enzymes were also evaluated to analyze the hepatotoxicity in our animal model.

## 2. Materials and Methods

### 2.1. Animals

All animal experiments were conducted in accordance with the guidelines of the ethics committee for use of animals in research at Yarmouk University (No. IACUC/2021/14). Male Balb/c mice (*n* = 10, 23–25 g) were individually housed in a controlled room with 21 ± 2 °C temperature, and 12:12 h light/dark cycle, with ad libitum access to food and water. Experimental groups (*n* = 5) were daily delivered an intraperitoneal injection of 3 mg/kg of synthetic cannabinoid XLR-11 (Cayman Chemical, Ann Arbor, MI, USA) dissolved in vehicle (dimethyl sulfoxide) for 5 consecutive days, the dose of the XLR-11 was selected based on literature [17,19]. The control group (*n* = 5) received daily injections of vehicle for the same period. On day 6, 24 h after the last injection, animals were sacrificed, blood samples were immediately collected for liver function tests, and liver tissues were collected for histological and gene expression analyses.

### 2.2. Biochemical Measures

The serum liver enzymes aspartate aminotransferase (AST) and alanine aminotransferase (ALT), and total protein were assayed using an immunoassay analyzer (ARCHITECT i1000SR, Abbott Laboratories, Chicago, IL, USA).

### 2.3. Histopathological Examination

Liver samples were fixed in 10% paraformaldehyde and processed for paraffin-embedding. Liver tissue blocks were sliced into 5 μm thick sections and stained with Hematoxylin and Eosin (H&E). H&E sections were evaluated for the structural changes of acute hepatic injury such as hepatocytes damage including different patterns of necrosis and immune cells infiltration.

In order to evaluate hepatic fibrosis, sections were stained with a Picro-Sirius Red Solution kit according to the manufacturer′s protocol (ab246832, Abcam, Cambridge, UK).

### 2.4. Total RNA Isolation and cDNA Synthesis

Total RNA extraction was performed from liver samples using a total RNA isolation kit (JenaBioscience, Jena, Germany), following the manufacturer’s instructions. RNA quantification and purity was revealed using the QuantiFluor RNA System (Promega, Madison, WI, USA) and Quantus Fluorometer (Promega, Madison, WI, USA).

Furthermore, for cDNA synthesis from RNA, first-strand cDNA was synthesized with 1 ug of total RNA using a Revert Aid First-strand cDNA synthesis kit (Thermo Fisher Scientific, Waltham, MA, USA) according to the manufacturer′s instructions. These samples were then frozen at −80 °C until real-time PCR was used to determine gene expression.

### 2.5. Quantitative Real-Time PCR (qPCR)

The expression levels of the mRNAs were defined using the quantitative real-time PCR (qPCR) technique. qPCR was carried out using the Line-Gene 9600 Real-Time PCR system (Bioer Technology, Bingjiang, China). The sets of primers (shown in Table 1) used for different genes were designed using the Primer 3 software (Whitehead Institute for Biomedical Research). The qPCR reaction was performed using the SYBR-PCR master- mix (FirePol qPCR Master Mix) according to the manufacturer′s protocol. The relative expression was calculated using the 2−∆∆Ct method. GAPDH gene was used as the internal reference to normalize the expression levels. Each experiment was performed in triplicate.

### 2.6. MDA Assay

Hepatic malondialdehyde (MDA) level, a marker of lipid peroxidation, was assessed according to the method of Buege and Aust [20].

### 2.7. TUNEL Assay

The terminal deoxynucleotidyl transferase-mediated dUTP-nick end labeling (TUNEL) assay was used to demonstrate the apoptotic cell death in the liver, which detects DNA fragmentation by labeling the 3′- hydroxyl termini in the double-strand DNA breaks generated during apoptosis. The staining was performed on paraffin-embedded liver sections using the in situ cell death detection kit (TUNEL assay, Abcam, Cambridge, MA, USA) according to the manufacturer′s instructions.

### 2.8. Statistical Analysis

GraphPad Prism was used for all statistical analyses (version 8.0.0 for Windows, GraphPad Software, San Diego, CA, USA). Data showed a normal (parametric) distribution. Data were expressed as mean +/− (SEM). Student’s *t*-test was used to compare the two tested groups.

## 3. Results

### 3.1. XLR-11 Treatment Induced Oxidative Stress, Inflammationy, and Apoptosis in the Liver

**Figure 1:**
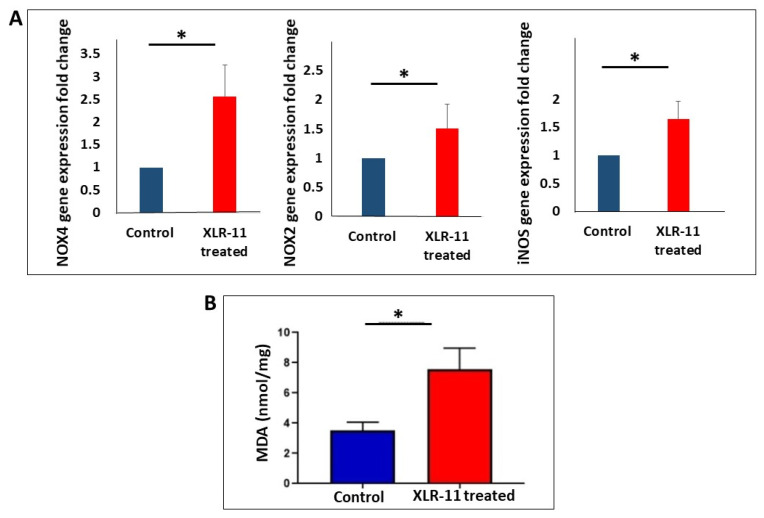
Gene expression of (**A**) markers of oxidative stress NOX4, NOX3, iNOS, and (**B**) MDA level in liver homogenate of control and XLR-11 treated mice, * *p*-value < 0.05.

We studied the effect of XLR-11 treatment on the expression of genes involved in oxidative stress, inflammation, and apoptosis in the liver using qRT-PCR. For oxidative stress, we investigated the mRNA expressions of NOX2, NOX4, and iNOS. As shown in Figure 1A, the expression of these three markers was significantly upregulated in the liver of XLR-11 treated mice compared to the control mice. Furthermore, MDA levels were assessed in XLR-11 treated livers to further evaluate the oxidative stress and the findings were in agreement with the qPCR results and revealed that XLR-11 treatment significantly induced MDA production compared to control group, (Figure 1B).

**Figure 2:**
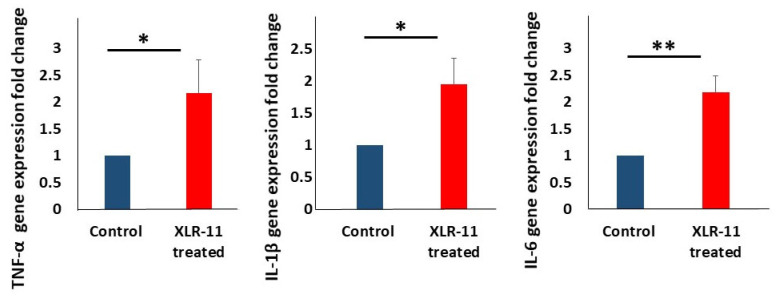
Makers of proinflammatory cytokines TNF-α, IL-1β, IL-6 in liver of XLR-11 treated mice compared to control mice, * *p*-value < 0.05, ** *p*-value < 0.005, respectively.

Similarly, the expression of the proinflammatory markers TNF-α, IL-1β, IL-6 were consistently elevated in the liver of XLR-11 treated mice compared to the control mice (Figure 2).

**Figure 3:**
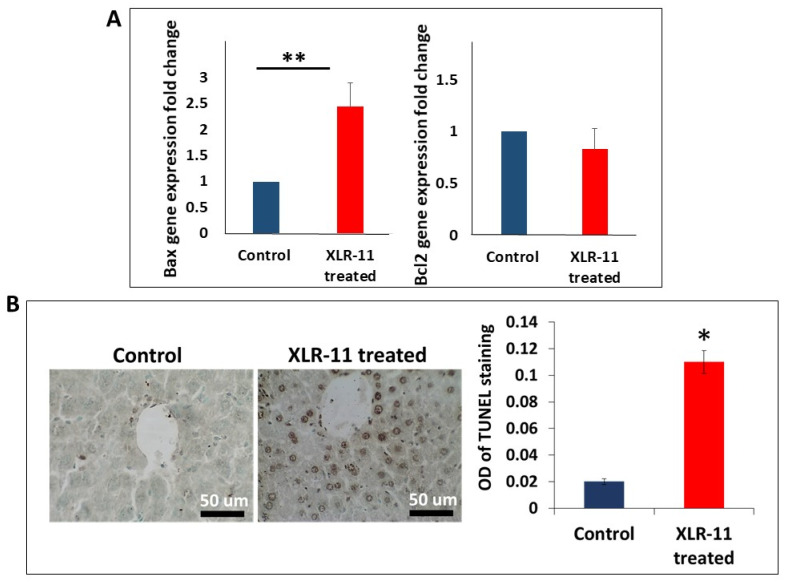
(**A**) Gene expression of markers of apoptosis Bax and Bcl2 in liver of XLR-11 treated mice compared to control mice, (**B**) TUNEL staining of the liver of XLR-11 treated mice compared to control mice, of * *p*-value < 0.05, ** *p*-value < 0.005, respectively.

For apoptosis, the expression of pro-apoptotic marker Bax was significantly upregulated in the liver of XLR-11 treated mice, whereas the expression of the anti-apoptotic marker Bcl2 showed no significant difference between the two groups (Figure 3A). This was further confirmed using TUNEL staining which revealed that the percentage of TUNEL-positive nuclei were significantly higher in XLR treated mice liver in comparison to control group (*p* < 0.05) (Figure 3B), which indicates apoptosis.

### 3.2. XLR-11 Treatment Induces Histopathological Changes in Liver

**Figure 4:**
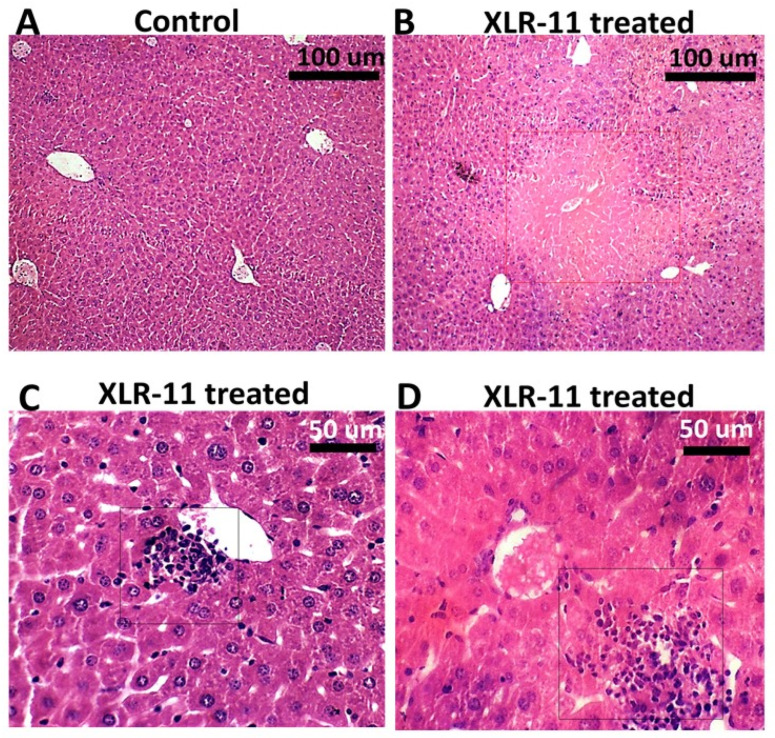
Histopathology of the liver from control mice (**A**) and XLR-11 treated mice (**B**–**D**). Red square box indicates centrilobular necrosis, black square boxes indicate spotty necrosis associated with lymphocytic infiltrate.

Liver tissues in XLR-11 treated mice were histopathologically evaluated and compared to the control group. As shown in Figure 4, the microscopic features of the examined sections show acute hepatic injury changes primarily active hepatocellular damage and necrosis. The necrosis ranges from spotty necrosis of minute clusters of hepatocytes associated with lymphocytic inflammation to larger areas of coagulative hepatic centrilobular necrosis within the centrilobular tissue of the hepatic lobule around the central vein typically in the acinar zone 3 area.

### 3.3. XLR-11 Treatment Alters AST and ALT Serum Level

**Figure 5:**
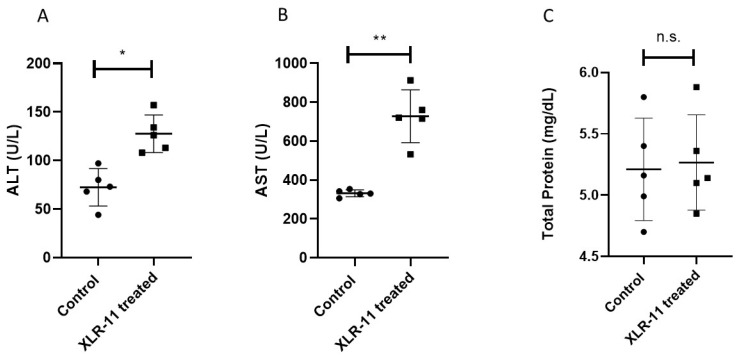
Levels of (**A**) ALT, (**B**) AST, and (**C**) total protein tests of liver function. * *p*-value < 0.05, ** *p*-value < 0.005, respectively, n.s. indicate no significance.

We analyzed the serum level of aminotransferases AST, ALT, and total protein in control mice and XLR-11 treated mice. Our results showed that XLR-11 treatment results in remarkable alteration of AST and ALT serum level (Figure 5A,B). The ALT was significantly higher in XLR-11 treated mice compared to control mice (+1.7-fold increase; *p* < 0.05). Similarly, significant increase of AST was found in XLR-11 treated mice (+2.1-fold increase; *p* < 0.005) compared to control mice. Finally, despite of lower total protein found in XLR-11 treated mice, no significant difference was found compared with control mice (Figure 5C).

### 3.4. XLR-11 Treatment Does Not Induce Fibrosis in Liver

**Figure 6:**
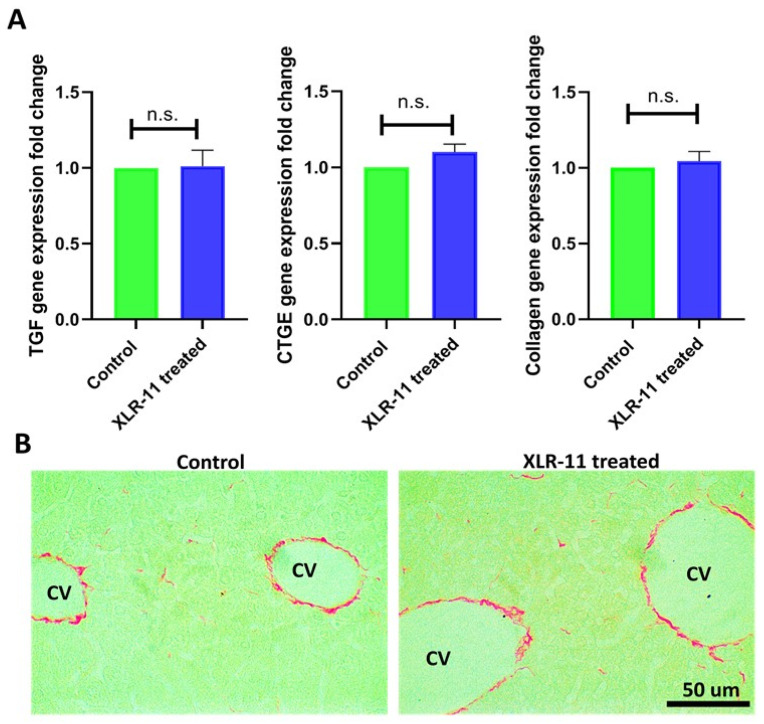
(**A**) Gene expression of fibrosis markers TGF-β, CTGF, Collagen 1 in liver of XLR-11 treated mice compared to control mice. (**B**) Picro-Sirius Red Stain of the liver from control mice and XLR-11 treated mice. Normal degree of fine liner red collagen fibers in the wall central veins in XLR-11 treated mice indicating no hepatic fibrosis; cv: central vein, n.s. indicate no significance.

In line with the histopathological assessment, we next evaluated the liver of XLR-11 treated mice for the presence of hepatic fibrosis. We first used qRT-PCR to analyze the expression of the fibrosis related genes TGF-β, CTGF, and Collagen 1 in the liver of XLR-11 treated and control mice. Our results showed no significant difference was found in the mRNA expression of these three genes between the two groups (Figure 6A). Furthermore, in using Picro-Sirius red stain, a special histochemical technique to visualize collagen, no considerable difference in the amount of collagen fibers was detected in the liver specimen of XLR-11 treated mice versus control mice (Figure 6B). Collectively, these results indicate that XLR-11 treatment in the time window used in this study is not implicated in the development of hepatic fibrosis.

## 4. Discussion

The increasing recreational use of SCs has become a severe health concern since reports of deaths and intoxication connected to SCs have significantly increased over the past decade. The usage of SCs in particular has been linked in multiple case studies to liver injury. In this regard, the literature has not yet provided a thorough evaluation of the toxic effect of SCs on the liver in vivo. The current study sought to investigate the potential toxicity of acute SC administration on liver tissue in a mouse model. Although it has not been specifically reported in the literature which synthetic cannabinoid substance caused liver injury, XLR-11 was chosen for this study because it is one of the most commonly consumed SCs. Several case reports of SC-related acute kidney injury and liver damage have been linked to XLR-11 and its parent compound, UR-144 [21]. Furthermore, XLR-11 has been also attributed to be the cause of death in postmortem case reports without pre-existing natural disease to explain death [22]. 

The metabolism of XLR-11 has been identified in several in vitro studies by using human hepatocyte cells, human hepatocellular carcinoma cells, pooled human liver microsomes, and recombinant human CYP enzymes. The major liver metabolites identified for XLR-11 were 2′-carboxy-XLR-11, UR-144 pentanoic acid, 5-hydroxy-UR-144, 2′-carboxy-UR-144 pentanoic acid, 2′-hydroxy-XLR-11 glucuronide, and 1′-hydroxy-XLR-11 glucuronide [23,24,25,26]. As previously demonstrated for other SCs, large numbers of these metabolites may retain significant affinity and activity at the CB1 and CB2 receptors [27,28,29]. Therefore, taking in account the high affinity and potency of SCs, together with their potential biotransformation to large numbers of highly active metabolites, suggest that SCs may have amplified effects when compared with the same level of exposure to THC. To the best of our knowledge, apart from their neurological effects, there are no studies focusing on the toxicological properties of SCs on liver in vivo. According to our finding, the acute administration of XLR-11 in mice markedly increased the mRNA expression of markers for oxidative stress, inflammation, and apoptosis in liver (Figure 1, Figure 2 and Figure 3). The induced gene expression of oxidative stress markers has been further confirmed by assessing the MDA levels in liver homogenate which showed significant increase in MDA levels, which indicates increased lipid peroxidation known to be associated with the oxidative stress [30]. On the other hand, TUNEL assay, which was developed to identify cells that have undergone apoptosis and are undergoing substantial DNA degradation [31], indicated the liver of the XLR-11 treated mice showed remarkable change in nuclei staining in TUNEL assay indicating apoptosis. In fact, it is now well known that high levels of SCs and their metabolites were reported to create oxidative stress that is mainly implicated in impairment of the function of mitochondria and endoplasmic reticulum [13,32,33,34]. This has been shown to be resolved in case reports of SC-related hepatotoxicity by treating patients with N-acetylcysteine, which acts to restore the oxidative capacity of the liver [13,16].

Along with oxidative stress, induction of inflammation was shown as a key mediator in SC-induced toxicity [35,36]. As a result of increased oxidative stress or by direct activation of the CB receptors, it is not clear which mode of action is potentially involved in increased inflammation in liver as a result of exposure to high concentration of SCs. However, an in vitro study on human lymphocytes showed that treatment of cells with the synthetic cannabinoids CP47 and 497-C8 increases in the induction of pro-inflammatory cytokines IL1, IL-6, and TNF-α [37]. Elevated TNFα levels may activate the transcription factor NFkB1, an important transcription factor involved in the up regulation of many other inflammatory mediators [38,39]. Increased infiltration of inflammatory cells may produce reactive oxygen species (ROS) and reactive nitrogen species (RNS) and further these can increase the expression of genes coding proinflammatory cytokines [40]. Therefore, based on our findings and previous studies, it is suggested that oxidative stress and inflammation are tightly interrelated and form a cruel cycle which is involved in the progression of deleterious cellular effects in the liver, leading to DNA damage and triggering apoptosis, which cause the progression to acute hepatic injury [13,37,41]. This is confirmed with pronounced hepatic necrosis and immune cell infiltration demonstrated in our histopathological examination in the liver of XLR-11 treated mice (Figure 4) as well as elevated liver enzymes ALT and AST in the serum of these mice (Figure 5A,B).

Whether the deleterious effect of XLR-11 on liver is mediated by secondary cytotoxic effects by high dose of XLR-11, or via direct activation of the CB1 and CB2 receptors within the liver, or both, is still not clear. Analysis of the genotoxic properties of XLR-11 in different experimental systems showed that XLR-11 is implicated in the induction of DNA damage but was considered unrelated to oxidative damage [42]. On the other hand, despite being found at low levels under usual physiological conditions, the CB1 and CB2 receptors have been shown to be expressed in various types of cells in the liver, including hepatocytes, hepatic myofibroblasts, vascular endothelial cells, and liver-resident immune cells [43,44,45], and were demonstrated to play a significant role in chronic liver diseases [46]. In addition, a study by Kim et al. showed that CB1 receptor is involved in the pathology of acute liver injury, and inhibition of the CB1 receptor in hepatocytes has been found to alleviate the inflammatory response to toxin-induced liver injury in animal model [47]. Therefore, it is reasonable to expect that XLR-11 may exerts its effect via direct activation of the CB1 and CB2 receptors within ECs in the liver, which contributes to the progression to acute liver injury.

It is worth mentioning that the fibrinogenic markers (collagen, TGF, CTGF) showed no significant change when comparing the XLR treated group with the control group (Figure 6); this could be attributed to the time window used in this study which is not implicated in the development of hepatic fibrosis [48].

Finally, it is important to report that in addition to the observed effect of XLR-11 on the liver tissue, some classical CNS symptoms of synthetic cannabinoid administration have been also observed, such as generalized seizers and decreased locomotor activity, especially within a few minutes from the administration of the drug, which lasts for 1–2 h upon each injection.

## 5. Conclusions

The results obtained from this study pointed out that acute administration of SCs can induce acute hepatic injury, which is seemingly mediated by activated oxidative stress and inflammatory processes in liver. Results of this study will be used as a ground base for further investigations to elucidate the molecular underlying mechanisms of SCs on liver at different doses and duration in both in vivo models and at clinical levels.

## Figures and Tables

**Table 1:** Primers’ sequences used for qPCR.

Gene	Forward Primer	Reverse Primer
NOX4	5′-TCATTTGGCTGTCCCTAAACG-3′	5′-AAGGATGAGGCTGCAGTTGAG-3′
NOX2	5′-CTGGTGTGGTTGGGGCTGAATGTC-3′	5′-CAGAGCCAGTGCTGACCCAAGGAGT-3′
iNOS	5′-ATGGACCAGTATAAGGCAAGC-3′	5′-GCTCTGGATGAGCCTATATTG-3′
TNF-α	5′-AAGCCTGTAGCCCACGTCGTA-3′	5′-AGGTACAACCCATCGGCTGG-3’
IL-1β	5'-AACCTGCTGGTGTGTGACGTTC-3'	5'-CAGCACGAGGCTTTTTTGTTGT-3'
IL-6	5'-ACAACCACGGCCTTCCCTACTT-3'	5'-CACGATTTCCCAGAGAACATGTG-3'
Bax	5′-CTGAGCTGACCTTGGAGC-3′	5′-GACTCCAGCCACAAAGATG-3
Bcl2	5′-GTGGATGACTGAGTACCT-3′	5′-CCAGGAGAAATCAAACAGAG-3′
TGF-β	5′-TCTACAACCAACACAACCCGG-3′	5′-GAGCGCACAATCATGTTGGAC-3′
CTGF	5’-CAAAGCAGCTGCAAATACCA-3’	5’-GGCCAAATGTGTCTTCCAGT-3’
Collagen 1	5′-TGGCCTTGGAGGAAACTTTG-3′	5′-CTTGGAAACCTTGTGGACCAG-3′

## Data Availability

Not applicable.
